# 

**Published:** 1860-04

**Authors:** 


*Maryland and Virginia Medical and Surgical Journal.*—The Virginia Medical and Surgical Journal has now two offices of publication, Richmond and Baltimore. The Richmond editorship remains, as before, in the hands of Dr. McCaw. The principal Baltimore editor is Dr. Chew Van Bibber. Knowing the latter gentleman as we do, we can sincerely offer our hearty congratulations to the subscribers of the Journal upon their good fortune in obtaining his services. He has secured the co-operation of a number of medical gentlemen, well known for their devotion to science, and their literary and professional abilities. The recent numbers of the Journal prove that the editors are not only able, but willing to furnish their subscribers with a first class medical monthly, and we commend to the favorable consideration of all who wish to keep up with the progress of medicine.

*Societies.*—A stated meeting of the Pennsylvania Association of Dental Surgeons was held on the evening of December 6th, 1859. The subject of discussion was the Different Methods of Preparing and Using Gold Foil in Filling Teeth. It was participated in by many, and some excellent remarks were made. The same Society met again, January 17th, 1860, when Dr. J. Hayhurst read a paper, Diptheritic Pharyngitis; after this paper, dental instruments came up for discussion.

The Michigan Dental Association held a meeting on the evening of January 15th, at Detroit. Essays on Difficult Dentition, and on the Effect of Diseased Teeth and Gums on the General Health, were read.

The Indiana State Dental Association met January 3d, 1860. Many interesting subjects were discussed, and a scale of professional charges were recommended for adoption.

